# Democratizing molecular diagnostics and building national capacity through open reagents and open PCR

**DOI:** 10.1186/s12919-026-00411-2

**Published:** 2026-11-09

**Authors:** Renuka Gadde, Josefina Campos, Barbara Tornimbene, Prabha Desikan, Allison Grande, Shesheer Kumar, Akenji Blaise Mboringong, Zeeshan Sidiq, Julia Tuttle

**Affiliations:** 1 Clinton Health Access Initiative, Boston, MA USA; 2 World Health Organization Hub for Pandemic and Epidemic Intelligence, Berlin, Germany; 3 CPC Analytics, Berlin, Germany; 4 Indian Council of Medical Research, Bhopal Memorial Hospital and Research Centre, Bhopal, India; 5 Thermo Fisher Scientific, Washington, DC USA; 6 Huwel Lifesciences, Hyderabad, India; 7 National Public Health Laboratory, Yaoundé, Cameroon; 8 New Delhi Tuberculosis Centre, New Delhi, India

**Keywords:** Open PCR, Molecular diagnostics, Open reagents, Laboratory capacity, Pandemic preparedness

## Abstract

Access to affordable, quality-assured molecular diagnostics remains uneven across low- and middle-income countries (LMICs). The COVID-19 pandemic showed how dependent many national testing networks were on a small number of closed platforms. Open PCR platforms and openly distributable assays filled much of that gap, but much of the capacity deployed during the pandemic has since gone unused. Session 16 of the WHO Hub for Pandemic and Epidemic Intelligence Innovation Forum, co-hosted with the Clinton Health Access Initiative (CHAI), examined how open reagents and open PCR are being adopted, financed and sustained within national health systems. Contributions came from CHAI, two tuberculosis laboratory experts from India speaking in a personal capacity, the National Public Health Laboratory of Cameroon, Huwel Lifesciences and Thermo Fisher Scientific. Examples included the validation of open PCR kits for tuberculosis within India's national programme, local manufacturing of reagents and kits, and Africa CDC's evaluation and listing of open PCR assays during the mpox and Ebola outbreaks. The common message was that the technology has been shown to work. The remaining challenges are to position it within national diagnostic networks, to validate assays rapidly when new pathogens emerge, and to keep platforms and staff in routine use between emergencies.

## Introduction

Diagnostics are the entry point to both clinical care and public health surveillance, yet access to them remains one of the most persistent gaps in global health. The Lancet Commission on Diagnostics estimated that almost half of the world's population has little or no access to diagnostics, with the burden falling most heavily on LMICs, where infectious diseases are most prevalent [2]. For molecular testing, access has historically been shaped by disease-specific programmes built around closed platforms, in which instruments, cartridges and assays come from a single manufacturer. These systems have delivered major gains for HIV and tuberculosis, but they leave countries dependent on the product decisions and supply capacity of a few suppliers [3].

The COVID-19 pandemic tested that model. Where closed-platform assays arrived late or in limited quantities, many countries turned to open real-time PCR instruments and openly distributable assays from multiple suppliers, and scaled testing rapidly from a base of almost none [4]. The experience raised a question that remains unresolved: whether the instruments, trained staff and supplier base built during the emergency can be sustained and used for other priority diseases, both routinely and when the next outbreak occurs. Open-source reagents and open diagnostic platforms are one way to answer it. They lower costs, reduce single-supplier dependencies and allow countries to adapt assays to their own epidemiological needs [5, 6].

The Pandemic and Epidemic Intelligence Innovation Forum is a quarterly exchange platform convened by the WHO Hub for Pandemic and Epidemic Intelligence [1]. Its sixteenth session was held on 7 July 2026 and co-hosted with CHAI. Rather than discussing the technology in the abstract, the session focused on the experience of those who have made open PCR work in practice. Its objectives were threefold: to provide an overview of the open-source reagents landscape; to examine how open PCR has been introduced and scaled within national health systems, including the regulatory, institutional and operational conditions that have enabled or hindered its adoption; and to identify actionable lessons on supply chain sustainability, national programme integration and multi-stakeholder coordination to inform the broader scale-up of open reagent solutions. The WHO Hub and CHAI opened the session with framing presentations. These were followed by a panel with contributions from two tuberculosis laboratory experts from India, a senior tuberculosis microbiologist and a tuberculosis laboratory specialist, both contributing in a personal capacity, as well as the National Public Health Laboratory of Cameroon, Huwel Lifesciences and Thermo Fisher Scientific, and a discussion with participants.

## How the COVID-19 pandemic shaped the use of open molecular diagnostics

In a health emergency, diagnostics are the first link in the chain of detection and response. Without them, we cannot confirm cases, trace contacts or measure the spread of a new pathogen or strain. Every delay in testing widens the gap between the start of an outbreak and the response to it. Among diagnostic tools, molecular diagnostics play a particular role at the start of an emergency. Their high sensitivity and specificity make them the reference method for confirming cases, including early in infection [7]. Laboratory platforms can process large numbers of samples, so testing can grow with an outbreak [8], and multiplex assays can distinguish between pathogens that cause similar symptoms [9]. Assays can also be redesigned as a pathogen evolves and new strains emerge [10].

Open molecular diagnostics are testing systems in which assay designs, reagents and instruments are not tied to a single manufacturer, so laboratories can run tests from several suppliers, or develop and adapt their own, on the same equipment [5, 6]. When a new pathogen emerges, countries can therefore begin testing as soon as any supplier, or their own reference laboratory, has an assay ready, rather than waiting for the manufacturer of a closed platform to produce one.

The value of this flexibility became evident during the COVID-19 pandemic. CHAI has supported molecular testing in LMICs for more than two decades, first around centralized laboratory capacity and sample transport for HIV programmes, and later for tuberculosis and near point-of-care testing. COVID-19, CHAI noted, exposed a hard reality: these networks had been designed around specific disease programmes, with site connections, data systems and turnaround times built for particular diseases, and they relied on closed devices. Countries were left dependent on a small number of suppliers, some of which did not offer COVID-19 assays until months after open reagent providers had begun supplying them. Driven by country demand, open PCR became the main route to scaling up testing.

The results were substantial. Across LMICs, open PCR and distributable assays accounted for more than 25 million tests in the first ten months of the pandemic, starting from almost no testing [11]. This is a conservative estimate based on procurement data from the WHO COVID-19 Diagnostics Consortium, which excludes domestic procurement in China, India, Brazil and South Africa, as well as some donations. A 2020 CHAI assessment found more than 4,500 devices, with an estimated capacity of over 1.3 billion tests per year, deployed across more than 120 LMICs. On the supply side, more than 20 manufacturers of distributable PCR assays received WHO Emergency Use Listing and were approved for procurement by the Global Fund, WHO and UNICEF. Staff were able to run tests after remote training alone, which CHAI presented as evidence of the platforms' usability, a frequent concern about open systems. Distributable assays were also available earlier than many closed-system assays, and were often priced at less than half their cost.

Manufacturers were shaped by the same experience. In India, Huwel Lifesciences recalled that at the start of the pandemic around 80% of molecular diagnostic inputs, including vials, primers, probes and instruments, had to be imported. During COVID-19, Huwel responded by building domestic production across the workflow and supplied around 52 million tests within India, with in-house manufacturing bringing the cost per test down substantially. Thermo Fisher Scientific recalled that it was the largest supplier of COVID-19 molecular tests to LMICs through the WHO COVID-19 Diagnostics Consortium, leveraging its secure supply chain to scale manufacturing capacity from 1 to 20 million tests per week within two months.

## Sustaining the gains: routine use as preparedness

Countries saw the COVID-19 experience as a success to replicate for other diseases. In a 2021 assessment by CHAI [4], the reported motivation was the large installed footprint, the costs of US$2–5 per test and a wide base of motivated suppliers. They also saw opportunities to increase competition, secure supply for epidemic-prone diseases and promote local manufacturing.

Open PCR could indeed shift from emergency response to preparedness and, according to the WHO Hub, this would strengthen public health through four capacities [5, 6, 12]. Access comes from more suppliers and lower-cost production and procurement. Adaptability comes from assays that can be modified as pathogens evolve. Resilience comes from diversified sourcing, which reduces dependence on a single supplier. Capability comes from local development, validation, technology transfer and manufacturing. The end point is laboratory data flowing into public health intelligence systems.

Contributors described these capacities in practice. Thermo Fisher Scientific and the National Public Health Laboratory of Cameroon agreed that laboratories already running routine or surveillance assays on open platforms, with instruments maintained and proficient staff, can switch quickly to an assay for a new pathogen, whereas closed systems depend on a manufacturer's decision to develop one. The National Public Health Laboratory of Cameroon added that, in its experience, open platforms are also easier to use and leave more room for research, and that countries themselves should maintain them. Huwel Lifesciences described open RT-PCR as a scalable, low-cost platform technology. A single instrument can run multi-pathogen panels and confirmatory tests for infectious and genetic diseases, including within One Health programmes, as well as environmental surveillance and antimicrobial resistance testing, which keeps platforms active between emergencies.

These capacities are also being built through manufacturing and markets. On access, the senior tuberculosis microbiologist noted that open platforms avoid proprietary cartridge lock-in and allow COVID-19 assets to be reused. Thermo Fisher Scientific described direct purchasing at reduced prices, a ceiling price for end users agreed with its distributors, and a single bundled price per sample result for countries used to procuring closed systems that way. On resilience and capability, the senior tuberculosis microbiologist argued that supply chains become more fragile with distance, so the more local the manufacturing, the more robust the supply. Huwel Lifesciences now manufactures across the workflow, from plastics, primers and probes to enzymes, amidites and dNTPs, which it said both controls quality and supports decentralized testing. The National Public Health Laboratory of Cameroon explained that Africa CDC has made local manufacturing an explicit aim, because diagnostics for many of the continent's priority diseases are not priorities for manufacturers serving high-income markets.

## What is “open” in open molecular diagnostics?

Despite the strong argument for open PCR to be used more broadly, and the described benefits, the 2024 CHAI assessment [11] captured that, outside China, India and Indonesia, open PCR was used mainly for research and small volumes of COVID-19 testing. Only about 5% of available capacity was in use, and countries tested an average of roughly three diseases on open platforms. Three manufacturers accounted for about 80% of devices outside India and China. The barriers included unclear regulatory pathways, weak quality systems, limited financing, poor supply coordination (including access pricing and service contracts), and wider gaps in human resources, infrastructure and sample transport. The disappointment, CHAI said, was the absence of a sustained strategy to maintain the investments in infrastructure, training and supply once the acute phase of the pandemic had passed.

Interest nonetheless remained. Nineteen of the 20 countries assessed still wanted to use open PCR to complement their existing networks. Pilots had begun for HPV testing in Rwanda, respiratory testing in Zambia, and tuberculosis testing in South Africa and Vietnam. CHAI also reported that, since the 2025 cuts to United States development assistance, stakeholders have signalled stronger demand for resilient, lower-cost testing, although this has not been formally assessed.

Why, then, did capacity that performed so well during the pandemic, and that countries are still keen to adopt, prove so hard to keep in routine use? Part of the answer lies in what "open" actually meant in practice. During COVID-19, openness at the level of instruments and assays was enough to scale testing quickly, because emergency listing, pooled procurement and donor funding filled the gaps around it. Once those arrangements ended, countries found that the rest of the system was far less open. For other diseases, reagent supply, quality assurance, regulatory pathways, financing and service contracts either depended on a few suppliers or had not yet been built. The WHO Hub's framing helps explain this. It proposed that "open" be understood as a spectrum across the layers of a diagnostic system rather than as a label attached to a product [5, 6].

The first layer is knowledge: publicly available assay designs, protocols, primer and probe sequences, and the validation data behind them. The second is reagents: the ability to source or produce enzymes, controls, primers and master mixes from more than one supplier. The third is platforms: instruments, consumables and workflows that are non-proprietary or broadly compatible. The fourth is the wider ecosystem, including transferable know-how, quality systems, regulatory pathways, manufacturing and data interoperability. A system may be open at one layer and highly dependent at another. This is what many countries experienced after COVID-19: open instruments, but dependence or gaps in the reagents, quality systems and regulatory pathways needed to use them routinely. Openness at one layer can even go unrecognized. A laboratory scientist in Nigeria assumed that the Thermo Fisher instruments in their laboratory were closed systems and asked whether new equipment would be needed to adopt open PCR; Thermo Fisher Scientific clarified that those models are open and that no new purchase would be required.

Technical openness does not, however, automatically create diagnostic capacity. An open protocol or compatible instrument still has to move a long pathway: assay development, analytical validation, clinical evaluation, regulatory authorization, manufacturing and quality assurance, procurement and supply, laboratory delivery with quality management and external quality assessment (EQA), and finally integration into surveillance and response [5, 8, 13]. Each step has its own requirements, from reference materials and validation capacity to demand aggregation, forecasting, sustainable financing, workforce, sample referral and interoperability. The bottleneck, the WHO Hub argued, is less the PCR chemistry itself than the system needed to translate it into routine public health use.

The opportunity is therefore to build national diagnostic ecosystems that link technology and standards, regulation and quality, markets and financing, laboratory networks, workforce and data, rather than open PCR capacity alone. The goal is to attain diversified national systems able to adopt, adapt and deploy diagnostics when and where they are needed, and some countries and regions have begun to build the needed infrastructure.

## From validation to national programmes

India offered the session's most developed example of open PCR moving from emergency use into a routine national programme. The senior tuberculosis microbiologist described how the goal of India's National Tuberculosis Elimination Programme (NTEP) to eliminate tuberculosis by 2030 made reliance on a single closed-system pipeline impractical. In order to find a solution, the Indian Council of Medical Research (ICMR) set up a multicentric network of laboratories to start the clinical validation of open PCR kits. When the kits, most of them manufactured in India, passed validation against standard protocols, they were recommended to NTEP for deployment [14].

What made this strategy practical rather than aspirational, the senior tuberculosis microbiologist said, was the RT-PCR infrastructure built across the country during COVID-19. The instruments were already in place and staff already trained, so only refresher training was needed and the kits could be absorbed into the programme quickly. Several kits for detecting *Mycobacterium tuberculosis* have been validated and recommended, including kits produced by Huwel Lifesciences, and NTEP has shown genuine openness to using them alongside the closed systems that have been the programme's backbone. Kits that combine detection with rifampicin and isoniazid resistance testing are under validation, with field feasibility studies to follow.

The tuberculosis laboratory specialist argued that open RT-PCR for tuberculosis has reached an important stage, and described two evaluations. The first was an analytical evaluation of a multiplex open RT-PCR assay that detects *M. tuberculosis* complex, non-tuberculous mycobacteria, and resistance to rifampicin and isoniazid [15]. The second was a field feasibility evaluation of an NTEP-approved open RT-PCR platform under routine laboratory conditions, covering workflow integration, operational feasibility and user acceptability. Both showed encouraging performance, in the laboratory and under programmatic conditions. With a growing evidence base [14, 15], the question is no longer whether the technology works but where within national diagnostic networks it delivers the greatest value: at which laboratory tier, in which testing scenario, and how it should be integrated with existing molecular networks.

The senior tuberculosis microbiologist framed the ultimate value of this integration in terms of epidemic intelligence rather than cost alone. Decentralized testing across the country can feed real-time data on prevalence and drug resistance into NTEP surveillance. Built-in resistance detection could flag emerging multidrug-resistant and extensively drug-resistant tuberculosis trends early. And the reused COVID-19 RT-PCR network provides a surge-ready platform for future outbreaks of any pathogen, not only tuberculosis.

Validation in India is also extending beyond tuberculosis. Huwel Lifesciences reported that it has validated multiplex assays detecting dengue, chikungunya, Zika and malaria in a single sample, using an extraction-free protocol on a newly launched four-colour open RT-PCR system, with deployment following quality approval through the ICMR system. Multiplexing matters because these diseases share symptoms, circulate in the same areas and can cause outbreaks at the same time, so a single test that distinguishes between them supports both clinical management and surveillance. Testing that reflects the combination of diseases a region actually faces, rather than global market priorities, is also at the heart of Africa CDC's work.

## From the national to the regional perspective

The National Public Health Laboratory of Cameroon described its work through the Africa CDC Diagnostic Advisory Committee to improve access to diagnostics for the continent's priority diseases. Africa CDC lists around 18 diseases that frequently cause outbreaks in Africa, from anthrax and cholera to Lassa fever, Marburg virus disease, mpox and yellow fever. Because many of these are rare in high-income countries, commercial tests for them are often scarce. As the moderators noted, these regional priorities may not be shared by other regions, which makes a regional focus on outbreak diagnostics critical.

Africa CDC's process combines evaluation requests from manufacturers with active scanning of the diagnostics landscape. Partners such as the African Society for Laboratory Medicine (ASLM), US CDC and the Global Fund finance the evaluations. Clinical evaluations take place at centres of excellence across Africa that hold well-characterized clinical samples and the necessary technology. Tests that meet the target product profile are recommended to African Union Member States, supported by harmonized registration through the African Medicines Agency and regional regulatory bodies, and inform procurement by the African Union, partners and Member States. Most of this work concerns open PCR assays. Decentralized point-of-care testing may be the long-term ideal, but the existing molecular footprint in Africa consists mainly of open PCR platforms.

Africa CDC applied this process during the mpox emergency [16]. When it declared a Public Health Emergency of Continental Security in August 2024 [16], there were not enough PCR assays for mpox, and most available products were for research use only. Clinical evaluations were carried out to bring them to a level suitable for emergency use, and several, including products from manufacturers in Egypt, Morocco, South Africa and South Korea, met the WHO target product profile. Member States, and Africa CDC itself, procured on the basis of the resulting list [16]. The same approach was under way for the Ebola outbreak at the time of the session, with three or four PCR assays identified and clinical evaluations in progress.

The Indian and African pathways have features in common. Both rely on independent evaluation at reference institutions with access to well-characterized samples, and both link that evaluation directly to a body that can act on it, whether a national programme or continental listing and procurement. Together they show how the ecosystem layer can be built in practice.

## Still work to be done

Despite this progress, contributors were clear about what remains to be done, both in routine programmes and in outbreak response. The senior tuberculosis microbiologist noted that internal and external quality control frameworks for open kits had to be built from scratch and must be in place before national deployment. Open systems demand greater technical skill and biosafety discipline than closed ones, reagent quality and supply reliability vary across manufacturers, and regulatory and policy alignment is needed for national scale-up. She added that regulatory hurdles had once been immense, but that ICMR had gone a long way towards harmonizing requirements and fast-tracking validation, so that the national programme can run its own feasibility studies and deploy kits in the field.

The tuberculosis laboratory specialist identified the barriers that remain once a product has regulatory approval and reaches the market. The first is strategic positioning: many tests are already approved, but where they belong in the network and what they are expected to achieve is still unclear. The second is implementation readiness, covering laboratory infrastructure, trained personnel, quality management systems, contamination control, and internal and external quality assessment. The third is standardization of interpretation, reporting and quality requirements across platforms.

His field study also raised a caution. Open RT-PCR systems had evolved naturally towards simplified workflows, through ready-to-use reagents, automated interpretation and dedicated software. These innovations add value, but the tuberculosis laboratory specialist warned against letting open systems become collections of proprietary workflows that resemble closed systems and lose the characteristics that make them open. Instead, he called for harmonized implementation guidance for open RT-PCR, including standardized approaches to result interpretation and reporting, quality management and competency assessment. This would bring consistency across platforms while preserving their flexibility.

In outbreaks, the main gap is the speed of validation. A participant asked why open PCR had seen limited use in the response to the Ebola disease outbreak caused by Bundibugyo virus, declared in the Democratic Republic of the Congo in May 2026 and also affecting Uganda [17]. The National Public Health Laboratory of Cameroon explained that this was because the reagents available for open platforms at the start of the outbreak could not detect the virus. The first assay that did was on a closed platform, so it became the main tool for case confirmation. Thermo Fisher Scientific added that many closed-system tests developed for Zaire ebolavirus after the 2014–2016 West Africa outbreak had also missed Bundibugyo virus, whereas it had a research-use-only assay that covered it, but without performance data it could not be deployed diagnostically at scale. In the United States, public health laboratories can use newly designed assays as laboratory-developed tests; Thermo Fisher Scientific described designing a hantavirus assay within 24 h and shipping it to public health partners within two days of design. Most laboratories in LMICs have no such option. Thermo Fisher Scientific proposed a rapid analytical and clinical validation process for open-system assays, and the moderator summed up the lesson: detecting a new virus is only half the task, and the other half is validating the assay quickly enough to deploy.

Workforce is a further condition, although the evidence here is more encouraging than the common perception that open systems are too complex for routine use. During COVID-19, staff could run open PCR tests after remote training alone, and in India only refresher training was needed for staff who had used the same instruments during the pandemic. The risk lies in what happens when platforms fall idle. As Thermo Fisher Scientific argued, workforce proficiency, like instrument service and maintenance, is maintained only when instruments are in regular use, and CHAI listed limited human resources and gaps in service contracts among the barriers behind low utilization.

Bringing the discussion together, the moderators summarized the panel's view as readiness combined with caution. Open PCR should not be allowed to become closed. Standardization and validation are needed so that quality is not compromised, and the necessary skills must be kept ready. Local production can ease supply problems. And a share of routine testing should run on open platforms, with countries deciding where and how, and regional bodies advising, so that capacity can be expanded quickly in an outbreak.

## The way forward

Closing the session, the WHO Hub noted that public health diagnostics remain a wide space for innovation, especially for detecting and responding to emerging infectious disease threats. The ongoing Ebola outbreak in the Democratic Republic of the Congo showed that strong national reference laboratories are necessary but not sufficient. Countries with remote areas also need diagnostic capability at the front end of their surveillance systems, and open reagents offer one route to it.

Across the session, participants agreed that the technical case for open PCR has been made. More than 25 million tests in the first months of COVID-19, validated tuberculosis kits in India and evaluated mpox assays in Africa all show that open platforms and distributable assays perform in LMIC settings. What the 2024 reassessment revealed is that capacity built in an emergency erodes without deliberate effort. Four priorities emerged from the discussion. First, countries need to decide where open PCR adds most value in their diagnostic networks, rather than treating it as a generic backup. Second, quality systems must keep pace, through harmonized guidance on interpretation, reporting, quality management and competency, supported by internal and external quality assessment, so that open systems stay open without becoming inconsistent. Third, supply needs to be diversified and brought closer to users, through local and regional manufacturing, transparent pricing, and bundled procurement and service models. Fourth, routine use should be maintained, keeping instruments in service and staff proficient so that platforms can pivot when an outbreak occurs.

The most concrete proposal concerned the gap between rapid assay design and deployment. The mpox and Ebola responses showed that open-system assays can be designed to detect a new pathogen or strain within days, yet cannot be deployed diagnostically without performance data. A coordinated rapid validation pathway for open-system assays could close that gap. It would bring together manufacturers, centres of excellence with characterized samples, regional bodies such as Africa CDC and the African Medicines Agency, and WHO. Building on the evaluation model Africa CDC has already applied during the mpox and Ebola responses, such a pathway would allow the open PCR capacity that countries already hold to be used from the earliest stage of the next outbreak.

## Data Availability

Not applicable.

## Abbreviations

Africa CDC: Africa Centres for Disease Control and Prevention
ASLM: African Society for Laboratory Medicine
CHAI: Clinton Health Access Initiative
COVID-19: Coronavirus disease 2019
EQA: External quality assessment
HIV: Human immunodeficiency virus
HPV: Human papillomavirus
ICMR: Indian Council of Medical Research
LMICs: Low- and middle-income countries
NTEP: National Tuberculosis Elimination Programme
PCR: Polymerase chain reaction
RT-PCR: Real-time (reverse transcription) PCR
UNICEF: United Nations Children's Fund
US CDC: United States Centers for Disease Control and Prevention
WHO: World Health Organization

## References

[1] Tornimbene B, Leiva Rioja ZB, Morgan O. Pandemic and epidemic intelligence innovation forum: bridging gaps in epidemic intelligence through global collaboration. BMC Proc. 2025. 10.1186/s12919-025-00316-6. PMC11869449 40022045

[2] Fleming KA, Horton S, Wilson ML, Atun R, DeStigter K, Flanigan J, et al. The Lancet Commission on diagnostics: transforming access to diagnostics. Lancet. 2021;398(10315):1997–2050. 10.1016/S0140-6736(21)00673-5. PMC8494468 34626542

[3] Médecins Sans Frontières Access Campaign. Time for $5: GeneXpert diagnostic tests. Technical brief. Geneva: MSF; 2019. https://msfaccess.org/time-for-5.

[4] Clinton Health Access Initiative. Molecular diagnostic systems: global footprint and market potential. Unpublished report; 2021.

[5] Matute T, Nuñez I, Rivera M, Reyes J, Blázquez-Sánchez P, Arce A, et al. Homebrew reagents for low-cost RT-LAMP. J Biomol Tech. 2021;32(3):114–20. 10.7171/jbt.21-3203-006. PMC8730520 35027869

[6] Alcántara R, Peñaranda K, Mendoza-Rojas G, Nakamoto JA, Martins-Luna J, Del Valle-Mendoza J, et al. Unlocking SARS-CoV-2 detection in low- and middle-income countries. Cell Rep Methods. 2021;1(7):100093. 10.1016/j.crmeth.2021.100093. PMC8529268 34697612

[7] World Health Organization. Recommendations for national SARS-CoV-2 testing strategies and diagnostic capacities: interim guidance, 25 June 2021. Geneva: WHO; 2021.

[8] Muttamba W, O’Hare BA, Saxena V, Bbuye M, Tyagi P, Ramsay A, et al. A systematic review of strategies adopted to scale up COVID-19 testing in low-, middle- and high-income countries. BMJ Open. 2022;12(11):e060838. 10.1136/bmjopen-2022-060838. PMC9676418 36396316

[9] Ramanan P, Bryson AL, Binnicker MJ, Pritt BS, Patel R. Syndromic panel-based testing in clinical microbiology. Clin Microbiol Rev. 2017;31(1):e00024-17. 10.1128/CMR.00024-17. PMC5740973 29142077

[10] Wang R, Hozumi Y, Yin C, Wei GW. Mutations on COVID-19 diagnostic targets. Genomics. 2020;112(6):5204–13. 10.1016/j.ygeno.2020.09.028 PMC7502284 32966857

[11] Clinton Health Access Initiative. Perspectives on open PCR: experience from scaling and future outlook. Unpublished data presented at the WHO Pandemic and Epidemic Intelligence Innovation Forum, Session 16; 7 Jul 2026.

[12] Cable J, Fauci A, Dowling WE, Günther S, Bente DA, Yadav PD, et al. Lessons from the pandemic: responding to emerging zoonotic viral diseases—a Keystone Symposia report. Ann N Y Acad Sci. 2022;1518(1):209–25. 10.1111/nyas.14898. PMC9538336 36183296

[13] Bashir IM, Mahmoud N, et al. Strengthening laboratories in response to outbreaks in humanitarian emergencies and conflict settings: results, challenges and lessons from expanding PCR diagnostic capacities for COVID-19 testing in Yemen. PLoS ONE. 2024;19(2):e0298603. 10.1371/journal.pone.0298603. PMC10889613 38394178

[14] Jayaprakasam M, Michael JS, Jain P, Basu S, Ajbani K, et al. Diagnostic accuracy of real-time PCR assay “Quantiplus® MTB FAST” for detection of adult pulmonary tuberculosis (PTB): a multi-centric study. Indian J Med Res. 2025;161(4):346–53. 10.25259/IJMR_767_2025. PMC12178193 40536373

[15] Sidiq Z, Anand A, Tyagi P, Dwivedi KK, Rajpal S, Chopra KK. Analytical performance of a multi-target open real-time PCR assay for simultaneous detection of tuberculosis, non-tuberculous mycobacteria, and drug resistance in a high-burden setting. Microbiol Spectr. 2026. 10.1128/spectrum.01643-26. PMC13689452 42603138

[16] Africa Centres for Disease Control and Prevention. Mpox molecular diagnostic tests (RT-PCR): recommendations as per 13 August 2024. Addis Ababa: Africa CDC; 2024. https://africacdc.org/download/mpox-molecular-diagnostic-testsrt-pcr/.

[17] World Health Organization. Ebola disease caused by Bundibugyo virus, Democratic Republic of the Congo & Uganda. Disease Outbreak News; 16 May 2026. https://www.who.int/emergencies/disease-outbreak-news/item/2026-DON602.

